# USP10 Is an Essential Deubiquitinase for Hematopoiesis and Inhibits Apoptosis of Long-Term Hematopoietic Stem Cells

**DOI:** 10.1016/j.stemcr.2016.11.003

**Published:** 2016-12-13

**Authors:** Masaya Higuchi, Hiroki Kawamura, Hideaki Matsuki, Toshifumi Hara, Masahiko Takahashi, Suguru Saito, Kousuke Saito, Shuying Jiang, Makoto Naito, Hiroshi Kiyonari, Masahiro Fujii

**Affiliations:** 1 Division of Virology, Niigata University Graduate School of Medical and Dental Sciences, 1-757 Asahimachi-Dori, Niigata 951-8510, Japan; 2 Department of Clinical Engineering and Medical Technology, Faculty of Medical Technology, Niigata University of Health and Welfare, Niigata 950-3198, Japan; 3 Division of Pathology, Niigata University Graduate School of Medical and Dental Sciences, Niigata 951-8510, Japan; 4 Niigata College of Medical Technology, Niigata 950-2076, Japan; 5 Animal Resource Development Unit and Genetic Engineering Team, RIKEN Center for Life Science Technologies, Kobe 650-0047, Japan

**Keywords:** hematopoietic stem cells, ubiquitin specific peptidase, apoptosis

## Abstract

Self-renewal, replication, and differentiation of hematopoietic stem cells (HSCs) are regulated by cytokines produced by niche cells in fetal liver and bone marrow. HSCs must overcome stresses induced by cytokine deprivation during normal development. In this study, we found that ubiquitin-specific peptidase 10 (USP10) is a crucial deubiquitinase for mouse hematopoiesis. All USP10 knockout (KO) mice died within 1 year because of bone marrow failure with pancytopenia. Bone marrow failure in these USP10-KO mice was associated with remarkable reductions of long-term HSCs (LT-HSCs) in bone marrow and fetal liver. Such USP10-KO fetal liver exhibited enhanced apoptosis of hematopoietic stem/progenitor cells (HSPCs) including LT-HSCs but not of lineage-committed progenitor cells. Transplantation of USP10-competent bone marrow cells into USP10-KO mice reconstituted multilineage hematopoiesis. These results suggest that USP10 is an essential deubiquitinase in hematopoiesis and functions by inhibiting apoptosis of HSPCs including LT-HSCs.

## Highlights

• Systemic USP10-knockout mice develop bone marrow failure because of HSC depletion
• USP10 inhibits apoptosis of long-term HSCs in fetal liver
• USP10 inhibits cytokine deprivation-induced apoptosis of fetal liver HSPCs in vitro

## Teaser

In this article, Fujii, Higuchi, and colleagues show that systemic USP10-knockout (USP10-KO) mice develop bone marrow failure associated with marked reductions in long-term hematopoietic stem cells (LT-HSCs). USP10-KO fetal liver cells exhibit enhanced apoptosis of hematopoietic stem/progenitor cells (HSPCs) including LT-HSCs. USP10 inhibits cytokine deprivation-induced apoptosis of HSPCs in vitro, and this inhibition requires the deubiquitinase activity of USP10.

## Introduction

Hematopoietic stem cells (HSCs) are essential for hematopoiesis throughout life. HSCs are a rare population of hematopoietic cells, having capabilities of both self-renewal and differentiation to all lineages of hematopoietic cells of T, B, myeloid, and erythroid cells (Orkin and Zon, 2008). During mouse development, HSCs first appear in aorta-gonad-mesonephros and placenta on embryonic day 11 (E11) and then move to fetal liver (FL), residing there until just before birth (Mikkola and Orkin, 2006). HSCs also start to colonize bone marrow (BM) around E17.5. After birth, HSCs are predominantly maintained in BM throughout normal life. Movement, self-renewal, and differentiation of HSCs are controlled by interactions of HSCs with a specialized environment, which is referred to as a niche. In addition, cytokines secreted by niche cells, such as stem cell factor (SCF), thrombopoietin (TPO), and CXCL12, also regulate the activity of HSCs (Anthony and Link, 2014, Morrison and Scadden, 2014, Ugarte and Forsberg, 2013).

During normal development, cytokine availability fluctuates partly because HSCs have to move from one niche to another, such as from FL to BM (Mazo et al., 2011). HSCs need to overcome apoptosis induced by cytokine fluctuation during normal development (Kohli and Passegue, 2014). Factors controlling apoptosis of HSCs play critical roles in hematopoiesis (Oguro and Iwama, 2007). For instance, *Mcl-1* is an anti-apoptotic gene, which is highly expressed in HSCs and inducible by SCF. *Mcl-1* knockout (KO) in mice results in BM failure due to the depletion of HSCs (Opferman et al., 2005).

Ubiquitin-specific peptidase 10 (USP10) is a member of the ubiquitin-specific protease family of cysteine proteases. USP10 has been shown to act as an anti-stress factor under several stress conditions, including oxidative stress, heat shock, and viral infection (Takahashi et al., 2013a, Takahashi et al., 2013b). A functional defect in USP10 may be associated with cancer. USP10 deubiquitinates and stabilizes the tumor suppressor p53, and SIRT6 (Lin et al., 2013, Yuan et al., 2010). USP10 deubiquitinates IKKγ/NEMO, thereby inhibiting IKKγ-mediated nuclear factor κB (NF-κB) activation after genotoxic stress (Niu et al., 2013). USP10 is downregulated in several highly aggressive renal clear cell carcinomas, and the downregulation is proposed to be a causative factor for cancer progression caused by reducing p53 protein stability (Yuan et al., 2010). Upon exposure to an oxidant, USP10 reduces production of reactive oxygen species (ROS), thereby inhibiting ROS-dependent apoptosis (Takahashi et al., 2013b). Analyses using USP10 mutants indicate that inhibition of ROS generation by USP10 does not require deubiquitinase activity (Takahashi et al., 2013b). Thus, USP10 has both deubiquitinase-dependent and -independent anti-stress functions.

In this study, we investigate USP10 function in vivo by generating USP10-KO mice. USP10-KO mice developed BM failure with severe anemia and died within 1 year. This BM failure with pancytopenia in USP10-KO mice was caused by the prominent reduction of hematopoietic stem/progenitor cells (HSPCs), especially long-term HSCs (LT-HSCs). USP10-KO FL HSPCs proliferated in the presence of the HSC cytokines SCF, TPO, FLT3 ligand, interleukin-3 (IL-3), and IL-6, equivalently to USP10 wild-type (WT) cells in vitro. Cytokine deprivation induced higher levels of apoptosis in USP10-KO cells, and the apoptosis was rescued by transduction of the USP10-WT gene but not by a deubiquitinase-defective mutant. Thus, USP10 is an essential deubiquitinase for mouse hematopoiesis and functions by inhibiting apoptosis of HSPCs including LT-HSCs.

## Results

### USP10-KO Mice Develop Bone Marrow Failure and Show Severe Anemia

We established systemic USP10-KO mice on a B6 genetic background (Figures S1A–S1D). USP10-KO mice were born at the expected Mendelian frequency (WT/Hetero [HET]/KO = 11:18:9). USP10-KO mice looked normal at birth, but within 1 day all nine USP10-KO mice died (data not shown). Thus, USP10 is essential for survival after birth.

**Figure 1:**
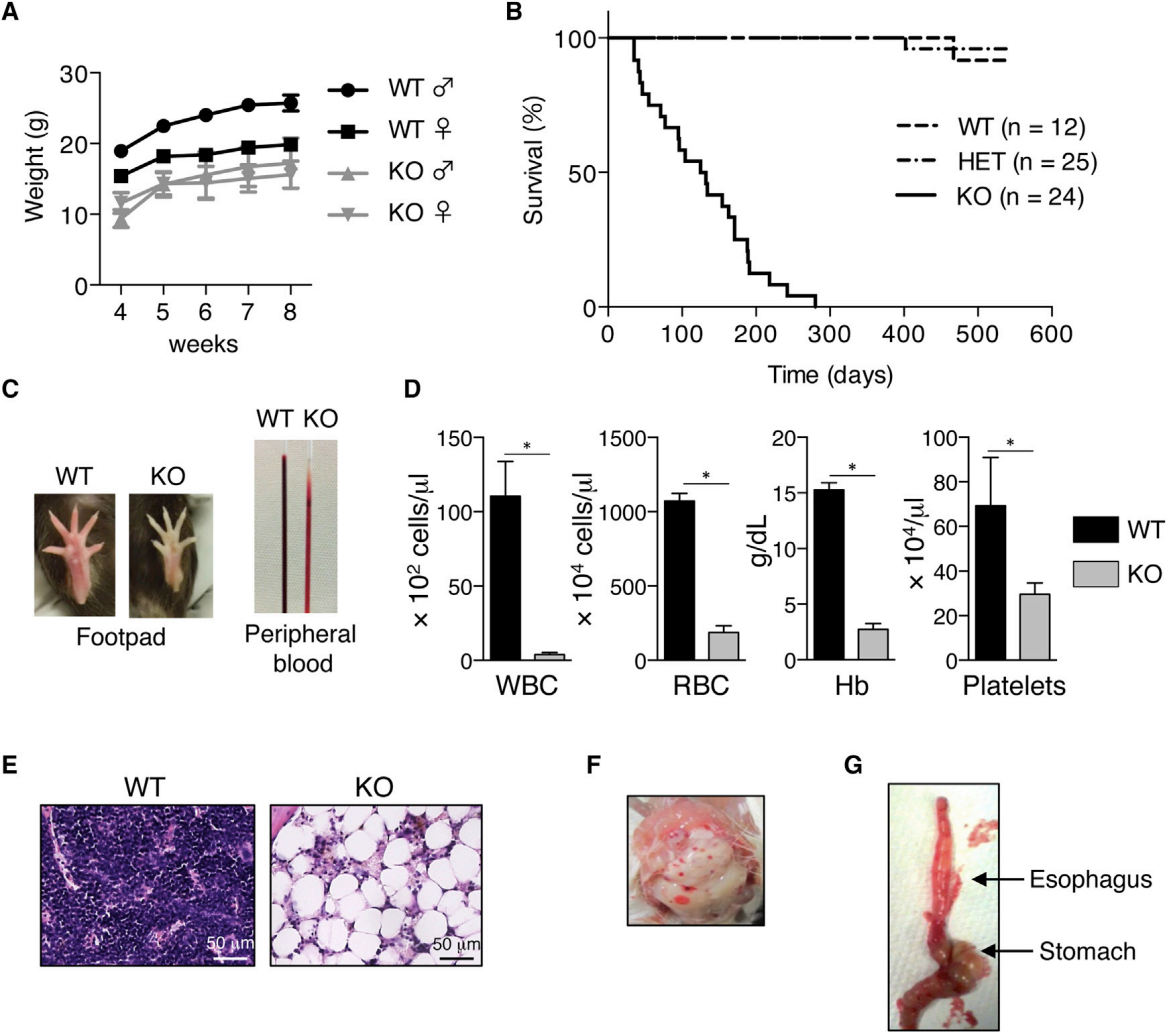
USP10-KO Mice Show Early Lethality and BM Failure (A) Growth retardation of USP10-KO mice. The body weights of male WT (n = 6), male KO (n = 5), female WT (n = 4), and female KO (n = 6) mice were monitored every week after 4 weeks of age. (B) Kaplan-Meier survival analysis of mice with the indicated genotypes. (C) Severe anemia in USP10-KO mice. The appearances of footpad and peripheral blood of a moribund USP10-KO mouse and those of an age-matched WT mouse are shown. (D) Blood cell counts from moribund USP10-KO mice and age-matched WT mice (mean ± SD; n = 4 for each genotype). ^∗^p < 0.05. (E) H&E staining of femur BM from 24-week-old USP10-WT and KO mice. (F and G) Cerebral and cerebellar hemorrhages (F) and esophageal achalasia (G) in USP10-KO mice.

Neonatal lethality in mice is often rescued by altering their genetic background. Thus, we established USP10-KO F2 hybrid mice with mixed genetic backgrounds, specifically B6 and BALB/c as described in Experimental Procedures. These USP10-KO F2 hybrid mice survived beyond the weaning period (4 weeks after birth), although the number of surviving USP10-KO mice was lower than that of USP10-competent mice (WT/HET/KO = 56:148:35). These USP10-KO mice were indistinguishable from USP10-WT mice at birth, but at around 2 weeks after birth they showed growth retardation (Figure 1A). In addition, at 5 weeks after birth some USP10-KO mice started to manifest several abnormalities including shallow breathing, scruffy fur coat, and lethargy. Within several days, these USP10-KO mice with abnormal manifestations inevitably became moribund. Within 300 days, all of the USP10-KO mice either died or were euthanized when they became moribund (Figure 1B). The onset of these abnormal manifestations in USP10-KO mice varied with regard to time. USP10-HET mice appeared healthy and survived longer than 300 days. Thus, USP10-HET mice and their cells were used as the WT samples in this study. Notably, all the moribund USP10-KO mice had pale footpads and their peripheral blood was anemic (Figure 1C). Peripheral blood collected from these moribund USP10-KO mice revealed a marked decrease in the number of white blood cells (WBCs) and red blood cells (RBCs), and in values for platelets and hemoglobin (Hb), relative to USP10-WT mice (Figure 1D). Pathological examination revealed that USP10-KO BM possessed a low number of nucleated cells and an increased amount of fat tissue (Figure 1E). This indicates that USP10-KO mice developed BM failure. In addition to BM failure, USP10-KO mice manifested two significant abnormalities, cerebral and cerebellar hemorrhaging (6 of 24) (Figure 1F) and esophageal achalasia (4 of 24) characterized by abnormally dilated esophagus overfilled with food and/or gas (Figure 1G). While male USP10-KO mice were fertile, the fertility of female USP10-KO mice remained to be determined (data not shown).

### Multiple Hematopoietic Lineage Defects Were Apparent in USP10-KO Mice

**Figure 2:**
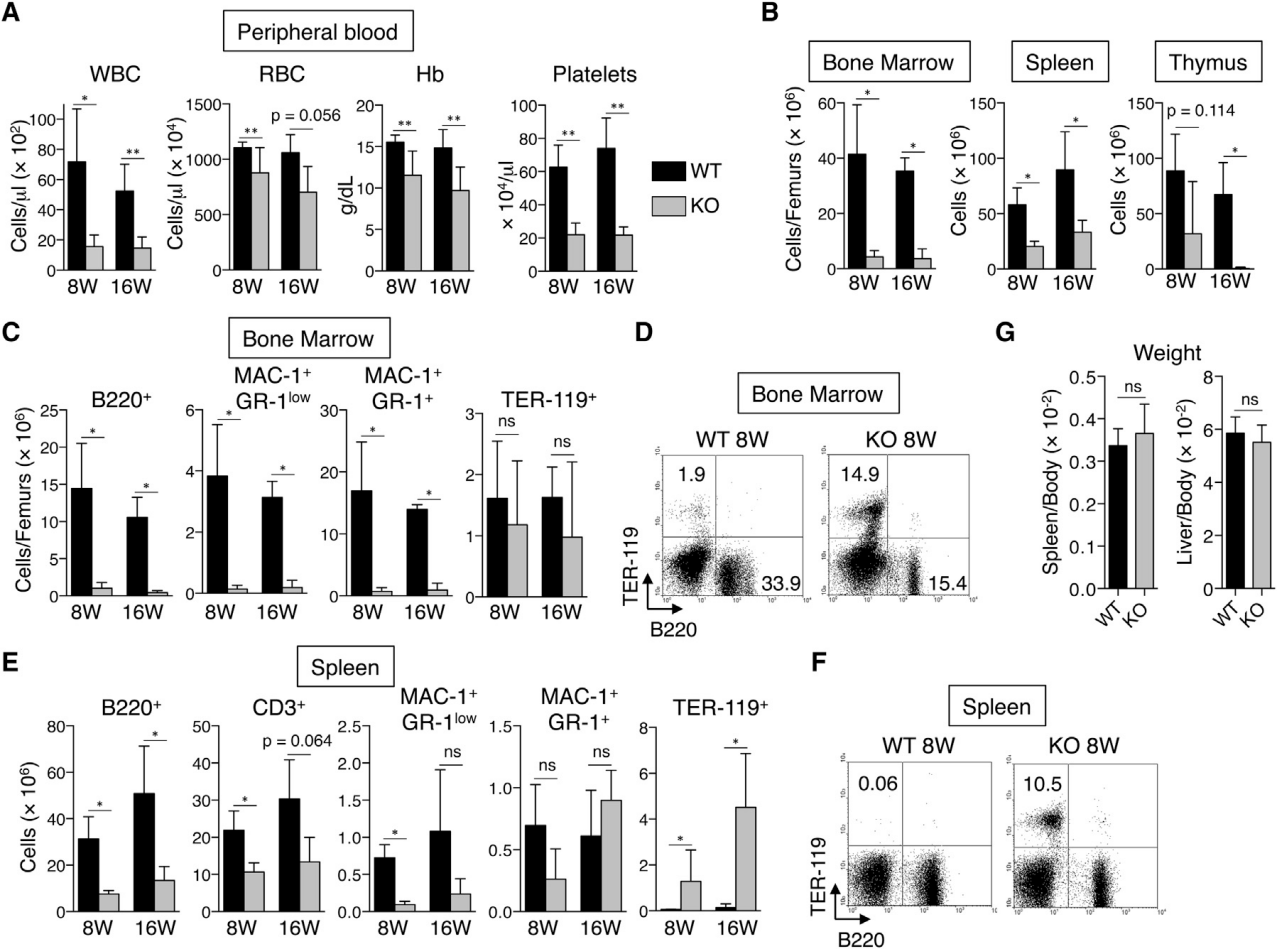
USP10-KO Mice Exhibit Pancytopenia and Multiple Hematopoietic Lineage Defects (A) Blood cell counts from 8- and 16-week-old USP10-WT and KO mice (mean ± SD; n = 5 for each genotype). (B) Cellularities of BM, spleen, and thymus from 8- and 16-week-old USP10-WT and KO mice (mean ± SD; n = 4 for each genotype except for n = 5 for 16-week WT). (C−F) BM (C and D) and spleens (E and F) were prepared from 8- and 16-week-old USP10-WT and KO mice, and then the numbers of B (B220^+^) and T (CD3^+^) cells, macrophages (MAC-1^+^GR-1^low^), granulocytes (MAC-1^+^GR-1^+^), and erythroid cells (TER-119^+^) in these organs were measured by fluorescence-activated cell sorting (FACS) analysis (mean ± SD; n = 4 for each genotype except for n = 5 for 16-week WT). Two femurs from each mouse were used to determine cell populations in BM. Numbers within the quadrants in (D) and (F) indicate the percentages of B220^+^ and TER-119^+^ cells. (G) Spleen and liver weights normalized by total body weight in 8-week-old USP10-WT and KO mice (mean ± SD; n = 5 and n = 4 for WT and KO, respectively). ^∗^p < 0.05; ^∗∗^p < 0.01. ns, not significant.

**Figure 3:**
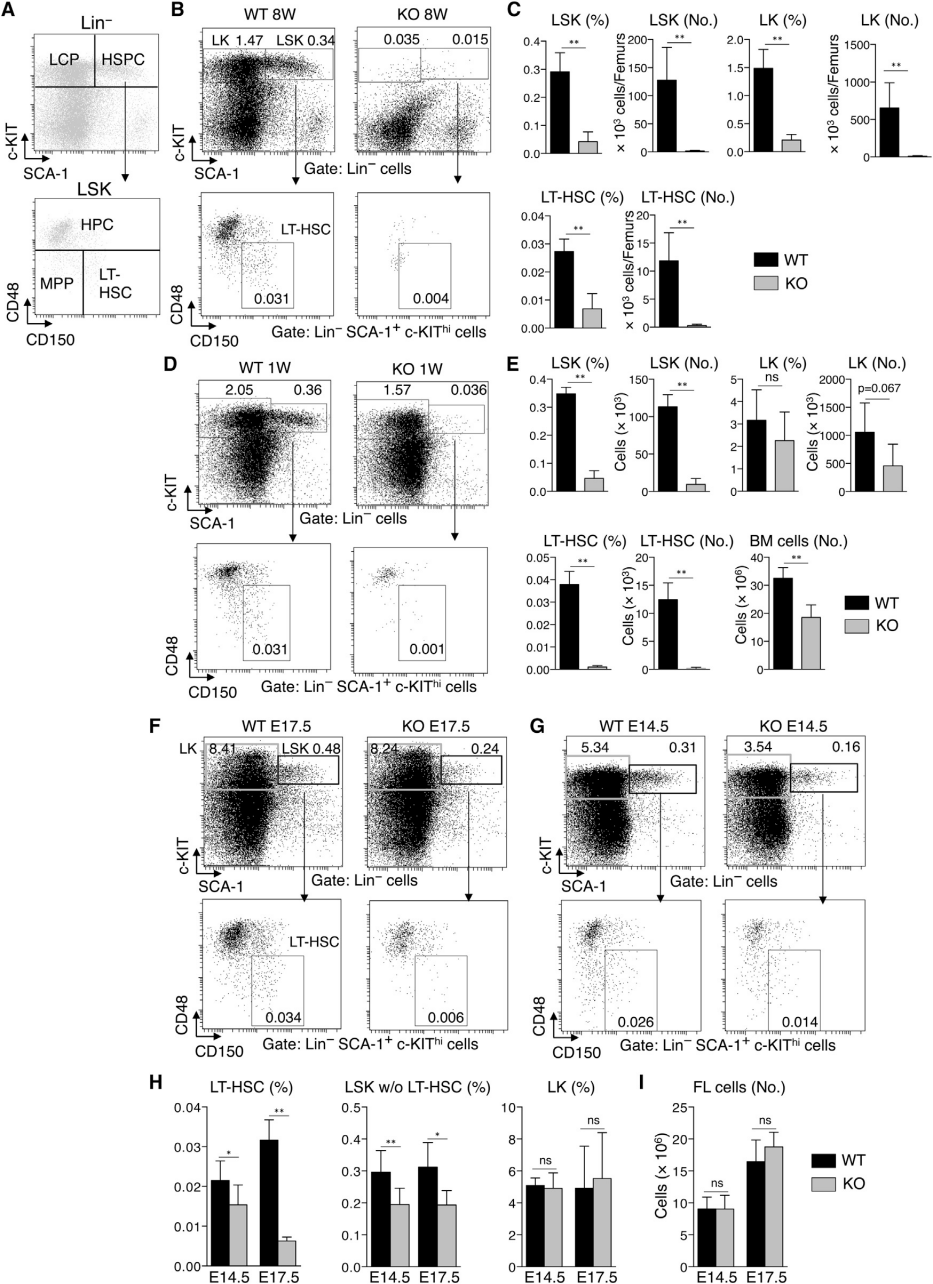
HSPCs Are Depleted in the BM and FL of USP10-KO Mice (A) Gating strategy for HSPCs. LK and LSK were labeled as LCP and HSPC, respectively (upper). CD48^+^ LSK, CD150^−^CD48^−^ LSK, and CD150^+^CD48^−^ LSK were labeled as HPC, MPP, and LT-HSC, respectively (lower). (B) Representative FACS analysis of BM cells from 8-week-old USP10-WT and KO mice comparing the frequencies of LK, LSK, and CD150^+^CD48^−^ LSK (LT-HSC) cells. Numbers indicate the percentages of each population. (C) Percentages and absolute numbers of LK, LSK, and LT-HSCs in the BM from 8-week-old mice (mean ± SD; n = 4 for each genotype). Two femurs from each mouse were used to determine cell populations. (D) Representative FACS analysis of BM cells from 1-week-old USP10-WT and KO mice comparing the frequencies of LK, LSK, and CD150^+^CD48^−^ LSK (LT-HSC) cells. (E) Percentages and absolute numbers of LK, LSK, and CD150^+^CD48^−^ LSK (LT-HSCs) in the BM from 1-week-old mice (mean ± SD; n = 4 for each genotype). (F and G) Representative FACS analysis of FL cells from E17.5 (F) and E14.5 (G) USP10-WT and KO embryos comparing the frequencies of LK, LSK, and CD150^+^CD48^−^ LSK (LT-HSC) cells. Numbers indicate the percentages of each population. (H and I) Percentages of CD150^+^CD48^−^ LSK (LT-HSCs), CD150^−^CD48^−^ and CD48^+^ LSK, and LK cells in FL cells (H) and absolute number of FL cells (I) from E14.5 and E17.5 USP10-WT and KO embryos (mean ± SD; n = 8, 9, 6, and 4 for E14.5 WT, E14.5 KO, E17.5 WT, and E17.5 KO, respectively). ^∗^p < 0.05; ^∗∗^p < 0.01. ns, not significant.

BM failure suggests that USP10-KO mice have impaired hematopoiesis. Stem cell antigen 1 (SCA-1) is widely used to characterize mouse hematopoiesis. However, it is expected that HSCs from one-quarter of the B6/BALB/c F2 hybrid do not express SCA-1, since BALB/c cells do not express SCA-1 (Spangrude and Brooks, 1993). Thus, we obtained USP10-KO B6/BALB/c F1 hybrid mice as described in Experimental Procedures. BM cells in USP10-KO F1 hybrid mice invariably expressed SCA-1 (Figure 3B), and were used for subsequent characterization. We characterized only USP10-KO F1 hybrid mice that appeared healthy. Peripheral blood collected from 8- and 16-week-old USP10-KO F1 hybrid mice showed a significant decrease in the number of WBCs and RBCs, and in values for platelets and Hb (Figure 2A). Like peripheral blood, the cellularity in BM, spleen, and thymus in USP10-KO mice markedly decreased (Figure 2B). In USP10-KO BM the numbers of B220^+^ B cells, MAC-1^+^GR-1^low^ macrophages, and MAC-1^+^GR-1^+^ granulocytes were markedly decreased (Figure 2C). Notably, the number of B220-low immature B cells in USP10-KO BM decreased more dramatically than B220-high mature B cells, suggesting that the decrease of immature B cells in USP10-KO BM precedes that of mature B cells (Figure 2D). The numbers of B cells, T cells, and MAC-1^+^GR-1^low^ macrophages were lower in USP10-KO spleen than in USP10-WT spleen. The decrease of MAC-1^+^GR-1^+^ granulocytes in USP10-KO spleen was not significant (Figure 2E). While the absolute number of TER-119^+^ erythroid cells in USP10-KO BM was equivalent to that of USP10-WT (Figure 2C), the proportion markedly increased in USP10-KO BM relative to USP10-WT BM. This was due to the decrease in the total number of hematopoietic cells in the KO group (Figure 2D). In addition, TER-119^+^ erythroid cells were detected only in USP10-KO spleen but not in USP10-WT spleen (Figures 2E and 2F). These results indicate that compensatory extramedullary hematopoiesis occurs in USP10-KO spleen, and complements the number of RBCs and Hb in USP10-KO mice (Figure 2A), but this compensatory system eventually breaks down, leading to severe anemia and early lethality in USP10-KO mice. Despite extramedullary hematopoiesis in the spleen, USP10-KO and USP10-WT mice did not exhibit differences between the weights of spleens or livers after normalization by total body weight (Figure 2G).

T cell differentiation marker analysis showed that the proportions of CD4, CD8, and CD4/CD8 double-positive cells in 8-week-old USP10-KO thymic tissue were equivalent to those in USP10-WT thymic tissue (Figure S2A). In addition, in vitro differentiation rates of USP10-KO FL c-KIT-positive progenitor cells into B cells and myeloid cells were equivalent to those of USP10-WT cells (Figure S2B). These results suggest that defective differentiation of these hematopoietic cells does not contribute to the pancytopenia observed in USP10-KO mice.

### HSC Depletion in USP10-KO Mice

The impaired hematopoiesis observed in USP10-KO mice prompted us to characterize HSCs and hematopoietic progenitor cells (HPCs) in this group. In adult BM, Lin^−^c-KIT^hi^ (LK) cells contain myeloid and erythroid lineage-committed progenitor cells (LCPs). Lin^−^SCA-1^+^c-KIT^hi^ (LSK) cells contain immature HPCs, multipotent progenitors (MPPs), and HSCs, and are defined as HSPCs (Figure 3A). CD48^+^ LSK, CD150^−^CD48^−^ LSK, and CD150^+^CD48^−^ LSK are defined as HPCs, MPPs, and LT-HSCs, respectively (Oguro et al., 2013). In 8-week-old mice the numbers of LK, LSK, and CD150^+^CD48^−^ LSK cells in USP10-KO BM were all dramatically decreased (1.51%, 1.28%, and 2.32% of USP10-WT, respectively) (Figures 3B and 3C). In 1-week-old mice, the numbers of LSK and CD150^+^CD48^−^ LSK cells in USP10-KO BM were also dramatically decreased (8.29% and 1.7% of USP10-WT, respectively) (Figures 3D and 3E), whereas the decrease in LK cells was statistically insignificant (Figure 3E). These results suggest that the decrease in CD150^+^CD48^−^ LSK (LT-HSCs) and LSK cells (HSPCs) in USP10-KO BM precedes that of more mature LK cells (LCPs).

To identify the onset of the HSPC decrease including LT-HSCs in USP10-KO mice, we characterized HSPCs in FL. In USP10-KO FL at E14.5, the frequencies of CD150^+^CD48^−^ LSK and LSK cells other than CD150^+^CD48^−^ LSK decreased to 72% and 66% of USP10-WT, respectively, and the decrease proceeded to 20% and 62% at E17.5 (Figures 3F–3H). It is noteworthy that the decreased level of CD150^+^CD48^−^ LSK cells (LT-HSCs) in USP10-KO FL was more than that of CD48^+^ LSK cells (HPCs). The frequency of LK and the total number of FL cells in USP10-KO mice at E17.5 and E14.5 were not significantly different from those of USP10-WT mice (Figures 3H and 3I). Collectively, these results suggest that the decrease of LT-HSCs in USP10-KO mice is the primary cause of BM failure and precedes E14.5 in FL.

### USP10-KO HSCs Have Defects in Long-Term Hematopoiesis Reconstitution Activity

**Figure 4:**
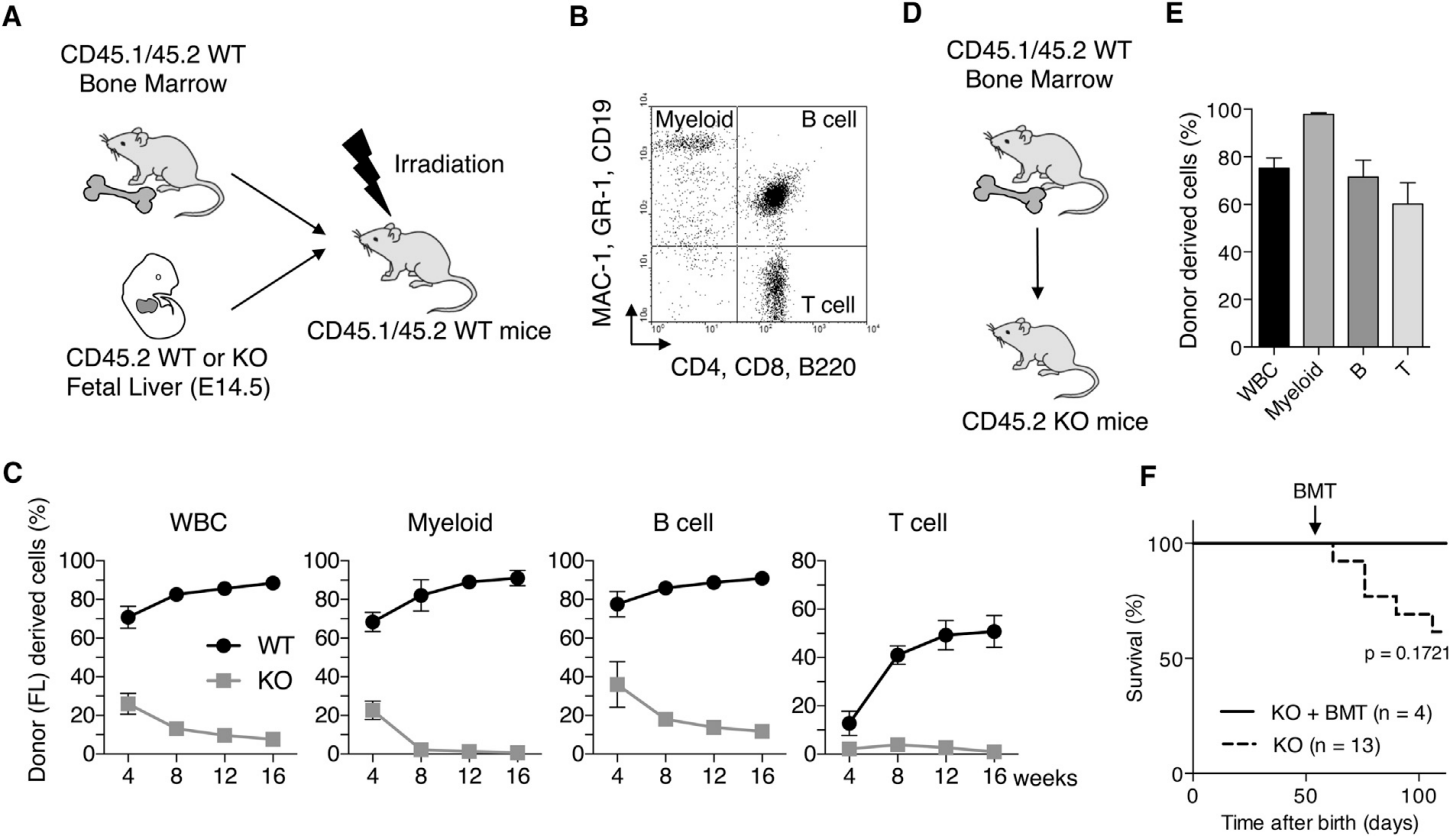
USP10-KO FL HSCs Have Defects in Hematopoietic Cell Reconstitution Activity (A) Experimental design of the competitive reconstitution assay. E14.5 USP10-WT or KO FL cells (CD45.2^+^) were mixed with WT BM cells (CD45.1^+^CD45.2^+^) and injected into lethally irradiated WT mice (CD45.1^+^CD45.2^+^). Reconstitution of peripheral blood cells by FL cells was monitored by CD45.1 staining for 16 weeks. (B) Representative FACS profile of peripheral blood cells. Myeloid-lineage (MAC-1^+^ or GR-1^+^), B-lineage (B220^+^ and CD19^+^), and T-lineage (CD4^+^ or CD8^+^) cells are shown. (C) Contribution of transplanted FL cells to peripheral WBCs, myeloid-, B-, and T-lineage cells in recipient mice (mean ± SD; n = 4 for each genotype). Data are representative of three independent experiments. (D) Experimental design of hematopoiesis rescue assay. USP10-WT BM cells (CD45.1^+^CD45.2^+^) were injected into 8-week-old USP10-KO mice (CD45.2^+^) and contribution of donor cells to peripheral blood cells was monitored by CD45.1 staining. (E) Contribution of donor BM derived cells to myeloid-, B-, and T-lineage cells 6 weeks after injection were measured (mean ± SD; n = 4). (F) Survival curves of USP10-KO mice with or without WT bone marrow transplantation (BMT). p Value was calculated by log-rank test.

To examine whether reduced HSC activity in USP10-KO mice is caused by defects in HSCs or by the environment supporting HSC activity, we performed an in vivo competitive long-term hematopoiesis reconstitution assay. CD45.1^+^CD45.2^+^ USP10-WT BM cells from young mice were combined with either CD45.2^+^ USP10-KO or USP10-WT FL cells at a 1:2 ratio, and the mixtures were transplanted into lethally irradiated CD45.1^+^CD45.2^+^ mice (Figure 4A). The reconstitution of myeloid, B, and T cells was examined by cell-surface staining of peripheral blood cells (Figure 4B). Four weeks after transplantation, USP10-WT FL cells reconstituted 70% of WBCs in peripheral blood of recipient mice, and at 16 weeks the reconstitution level increased to 88% (Figure 4C). In contrast, 4 weeks after transplantation, USP10-KO FL cells reconstituted only about 25% of WBCs in the recipient mice, and at 16 weeks the reconstitution decreased to 10%. USP10-KO FL cells showed reduced reconstitution of myeloid, B, and T cells relative to those of USP10-WT cells (Figure 4C). As an inverse experiment, CD45.1^+^CD45.2^+^ USP10-WT BM cells were transplanted into 7- to 9-week-old CD45.2^+^ USP10-KO mice (Figure 4D). At 6 weeks after transplantation, USP10-WT BM cells efficiently reconstituted hematopoiesis of WBCs and myeloid, B, and T cells in recipient USP10-KO mice (Figure 4E). Collectively, these results suggest a potential intrinsic defect in USP10-KO HSCs, which could cause the reduction of HSC numbers and the development of BM failure with pancytopenia. We measured the survival time of USP10-KO F1 mice with or without transplantation of USP10-WT BM cells (Figure 4F). While all four USP10-KO mice with USP10-WT BM cells lived longer than 100 days, 5 of 13 USP10-KO mice without USP10-WT cells died within 100 days, although the difference between these two groups was not statistically significant because of the small sample size and short observation period. Furthermore, one USP10-WT BM-transplanted USP10-KO mouse lived for 192 days and the other three lived for more than 300 days without any abnormal manifestations other than smaller body size relative to USP10-WT mice. These results suggest that BM failure is the main cause of early death seen in USP10-KO mice with mixed genetic background.

### USP10-KO HSCs Undergo Enhanced Apoptosis

**Figure 5:**
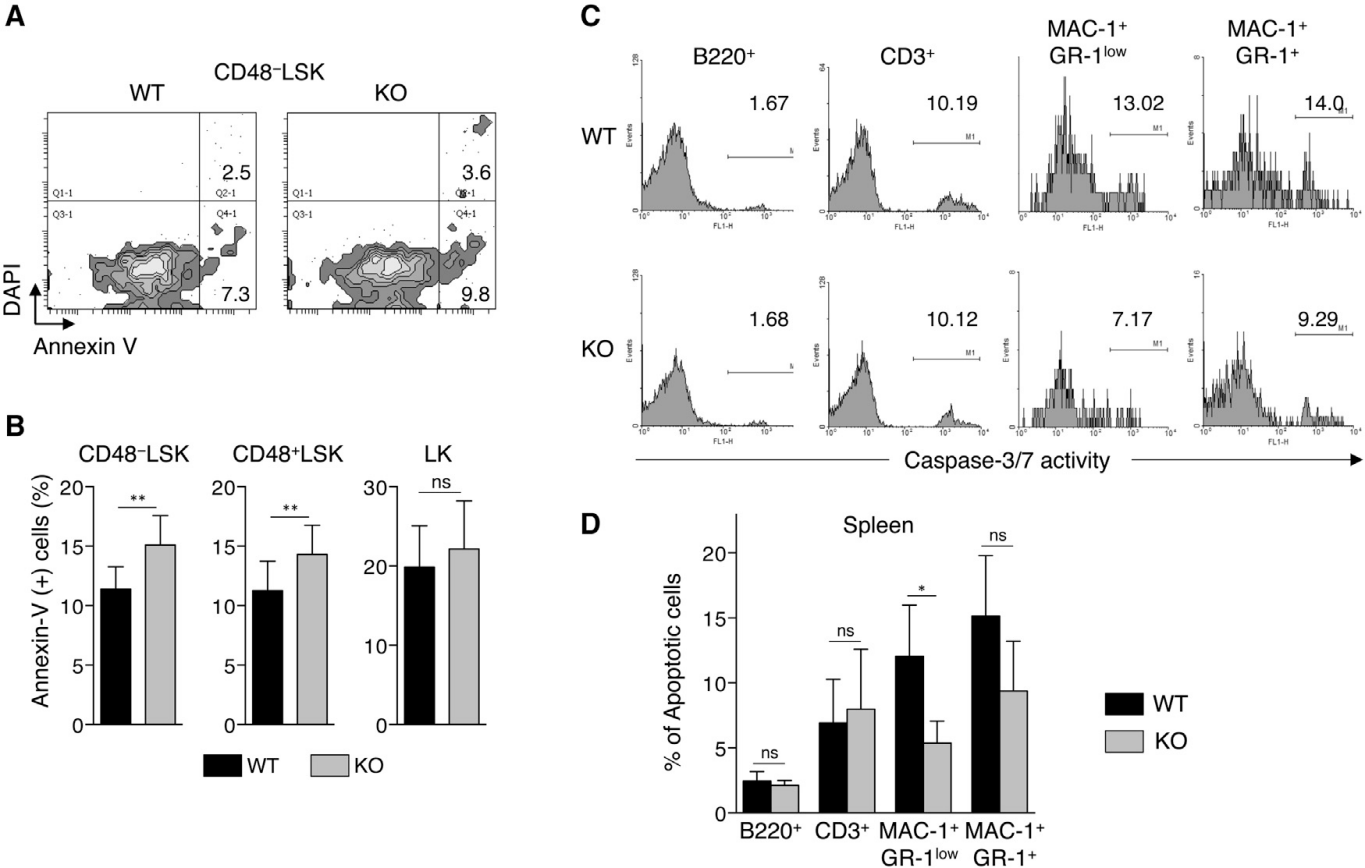
USP10-KO FL LSK Cells Show Enhanced Apoptosis (A and B) FACS analysis of apoptotic cells in CD48^−^ LSK, CD48^+^ LSK, and LK cells from E14.5 USP10-WT and KO FL. (A) Representative FACS analysis of CD48^−^ LSK cells stained with annexin V and DAPI. Numbers indicate the percentages of each population. (B) Percentage of annexin V-positive cells in CD48^−^ LSK, CD48^+^ LSK, and LK cells (mean ± SD; n = 15 and n = 13 for WT and KO, respectively). (C and D) FACS analysis of apoptotic cells in B220^+^, CD3^+^, MAC-1^+^GR-1^low^, and MAC-1^+^GR-1^+^ cells form 8-week-old USP10-WT and KO spleen. (C) Representative FACS analysis of caspase-3/7 activity from each population. Numbers indicate the percentage of apoptotic cells. (D) Percentage of apoptotic cells in each population (mean ± SD; n = 5 and n = 4 for WT and KO, respectively). ^∗^p < 0.05; ^∗∗^p < 0.01. ns, not significant.

To delineate how USP10 deficiency results in depletion of HSPCs including LT-HSCs in USP10-KO mice, we examined apoptosis of FL cells by staining with annexin V and DAPI. We detected an increase in apoptosis in USP10-KO CD48^−^ LSK (HSC/MPP) and CD48^+^ LSK (HPC) cells compared with USP10-WT cells (Figures 5A and 5B). In contrast to CD48^−^ LSK and CD48^+^ LSK, USP10-KO LK (LCP) cells showed apoptosis equivalent to that of USP10-WT cells. These results suggest that inhibition of HSC apoptosis is a function of USP10 to maintain the number of HSCs in FL, and that USP10 selectively inhibits apoptosis of HSCs, MPPs, and HPCs but not more differentiated LCPs. Additionally B, T, and myeloid cells prepared from 8-week-old USP10-KO spleens showed either equivalent or decreased levels of apoptosis relative to USP10-WT cells (Figures 5C and 5D). These results support our conclusion that selective apoptosis of USP10-KO HSPCs is a cause of the pancytopenia seen in USP10-KO mice.

### USP10-KO FL LSK Cells Are More Susceptible to Cytokine Deprivation-Induced Apoptosis

**Figure 6:**
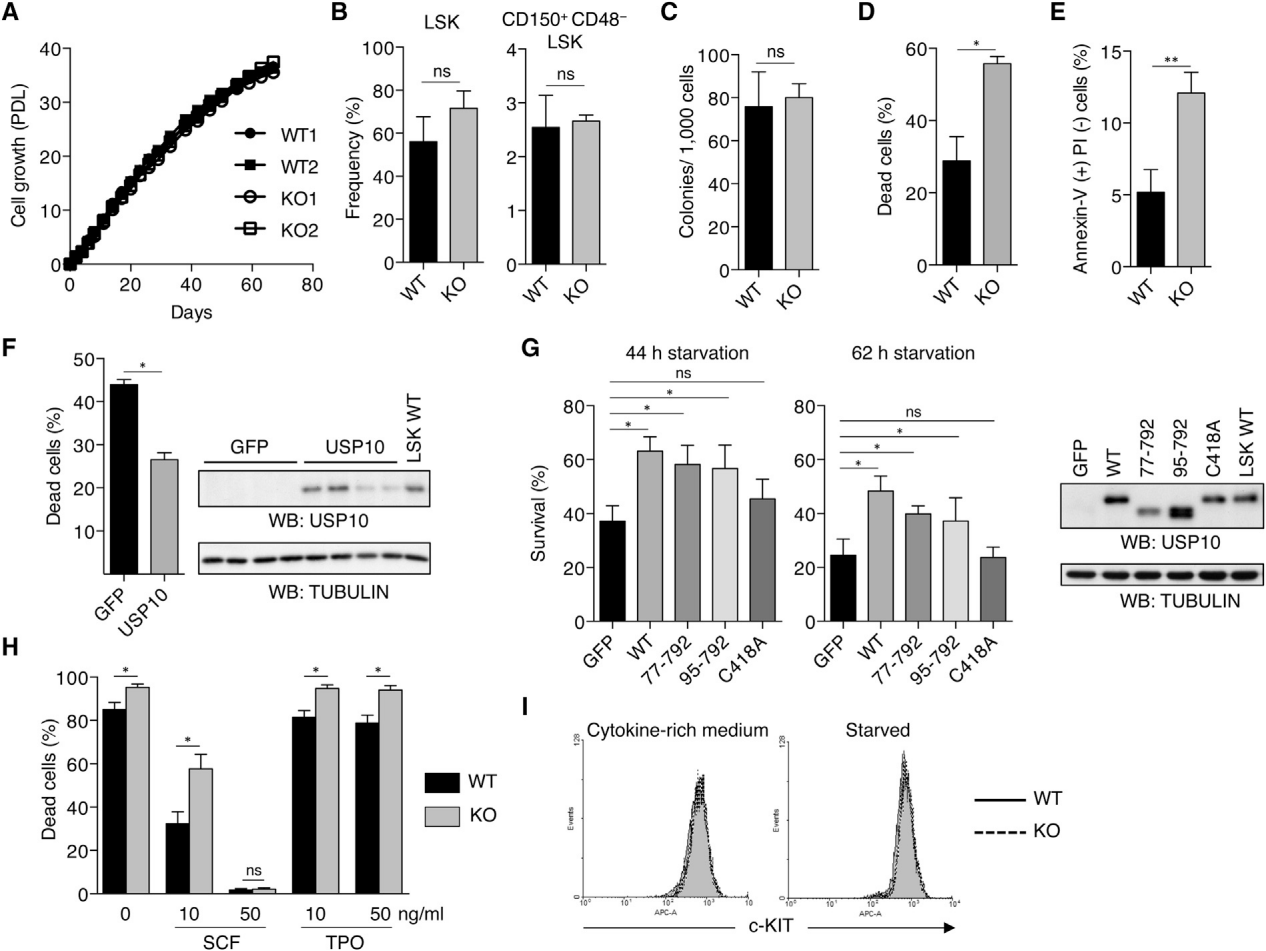
USP10-KO FL LSK Cells Are More Susceptible to Cytokine Deprivation-Induced Apoptosis In Vitro (A) In vitro growth of USP10-WT and KO FL cells isolated from E14.5 embryos. FL cells were cultured for more than 60 days in the presence of HSC cytokines with serial passages every 3 or 4 days. Cell number was determined by trypan blue staining, and the number of population doubling (PDL) is indicated. (B) Frequency of LSK and CD150^+^CD48^−^ LSK cells in FL cells after culturing with HSC cytokines for 1 week (mean ± SD; n = 4 for each genotype). (C) In vitro colony-formation capacity of USP10-WT and KO IVC-LSK cells (mean ± SD; n = 4 for each genotype). (D) USP10-WT and KO IVC-LSK cells were cultured in cytokine-low medium (1/50 concentration of HSC-cytokine medium) for 48 hr, and the cell viability was determined by trypan blue staining (mean ± SD; n = 4 for each genotype). At least 200 cells were counted for each sample. ^∗^p < 0.05. (E) USP10-WT and KO IVC-LSK cells were incubated in cytokine-free medium for 12 hr. After staining with annexin V and propidium iodide (PI), cells were analyzed by FACS (mean ± SD; n = 3 and n = 4 for WT and KO, respectively). ^∗∗^p < 0.01 by Student's t test. (F) USP10-KO IVC-LSK cells (n = 4) were transduced with lentiviruses encoding either GFP or USP10, and the cell viability after cytokine starvation for 44 hr was examined by trypan blue staining (mean ± SD). Total cell lysates from the transduced cells were analyzed by western blotting using anti-USP10 or anti-TUBULIN antibodies. Lysates from USP10-WT IVC-LSK were used as a control. At least 200 cells were counted for each sample. Data are representative of three independent experiments. ^∗^p < 0.05. (G) USP10-KO IVC-LSK cells were transduced with lentiviruses encoding GFP, USP10, USP10/77–792, USP10/95–792, or USP10/C418A, and the cell viability after cytokine starvation for 44 hr and 62 hr was examined by trypan blue staining. At least 250 cells were counted for each sample. Total cell lysates from the transduced cells were analyzed by western blotting using anti-USP10 or anti-TUBULIN antibody. Data are pooled from five independent experiments and presented as mean ± SD. ^∗^p < 0.05 by Tukey's multiple comparison test. (H) USP10-WT and KO IVC-LSK cells were cultured in 0, 10, or 50 ng/mL SCF or TPO for 24 hr and the cell viability was determined by PI staining and FACS analysis (mean ± SD; n = 4 for each genotype). At least 10,000 cells were counted for each sample. ^∗^p < 0.05. (I) Cell-surface c-KIT expression in USP10-WT and KO IVC-LSK cells in cytokine-rich medium or after starvation for 6 hr. “n” represents number of embryos from which IVC-LSK cells were prepared. ns, not significant.

Hematopoietic cells prepared from FL can grow and expand in the presence of growth-promoting cytokines, specifically SCF, TPO, FLT3 ligand, IL-3, and IL-6 (Sauvageau et al., 2004). We examined whether USP10 plays a role in cytokine-induced growth of FL cells. USP10-KO cells prepared from E14.5 FL continuously grew in the presence of HSC cytokines, and the growth rate was equivalent to that of USP10-WT FL cells, but after 2 months in culture both cell types stopped proliferating (Figure 6A). After USP10-KO FL cells were cultured with HSC cytokines for 1 week, the populations of LSK and CD150^+^CD48^−^ LSK cells were increased to 75% and 2.5% of total cells, respectively, and the increases were equivalent to those of USP10-WT cells (Figure 6B). We next examined in vitro colony-forming activity of FL LSK cells expanded by HSC cytokines. We first cultured FL cells with HSC cytokines for 1 week, and c-KIT-positive cells were purified using magnetic beads. After purification, more than 90% of c-KIT-positive cells were lineage negative and SCA-1 positive (LSK, data not shown), and we named these purified cells in vitro cultured LSK (IVC-LSK) cells. USP10-KO IVC-LSK cells produced an equivalent number of colonies in methylcellulose compared with those of USP10-WT IVC-LSK cells (Figure 6C). These results suggest that USP10-KO LSK cells prepared from FL undergo self-renewal in HSC cytokine-rich medium to an equal extent as USP10-WT cells in vitro.

Next, we studied cytokine deprivation-induced cell death of USP10-KO IVC-LSK cells. USP10-KO IVC-LSK cells were cultured in cytokine-low medium (1/50 concentration of HSC cytokine-rich medium) for 48 hr, and cell viability was examined by trypan blue staining. USP10-KO IVC-LSK cells exhibited significantly higher cell death compared with USP10-WT cells (Figure 6D). The cell death was induced by apoptosis detected by annexin V staining (Figure 6E). Transduction of the USP10-WT gene into USP10-KO IVC-LSK cells significantly inhibited cell death (Figure 6F). To determine which USP10 function inhibits cell death after cytokine starvation, we examined three USP10 mutants (Figure 6G). While USP10/77-792 and USP10/95-792 mutants inhibited cell death of IVC-LSK cells as efficiently as USP10-WT, a deubiquitinase-inactive mutant USP10/C418A showed minimal activity and failed to inhibit cytokine deprivation-induced cell death after 44 and 62 hr of starvation. We previously reported that USP10 inhibits oxidant-induced apoptosis by reducing ROS production in mouse embryonic fibroblasts (MEFs) (Takahashi et al., 2013b). Unlike cytokine deprivation-induced apoptosis of IVC-LSK cells, oxidant-induced apoptosis of MEFs was inhibited by USP10/C418A but not USP10/77–792 or USP10/95–792 (Takahashi et al., 2013b). The expression levels of mutant USP10 proteins in USP10-KO cells were equivalent to that of USP10-WT (Figure 6G). Collectively, these results indicate that USP10 inhibits cytokine deprivation-induced apoptosis of IVC-LSK cells in a deubiquitinase-dependent but ROS-independent manner.

The HSC cytokines used for in vitro culture of HSPCs were SCF, TPO, FLT3 ligand, IL-3, and IL-6. Of these, SCF and TPO are regarded as the most crucial cytokines for HSPC expansion and maintenance (Ding et al., 2012, Qian et al., 2007, Yoshihara et al., 2007). Therefore, we examined the ability of these two cytokines to inhibit the apoptosis of IVC-LSK cells using cytokine withdrawal experiments. SCF (50 ng/mL), but not TPO, almost completely inhibited apoptosis of both USP10-KO and USP10-WT IVC-LSK cells (Figure 6H). These findings indicate that SCF contributes to the survival of IVC-LSK cells by inhibiting apoptosis. However, decreasing the concentration of SCF from 50 ng/mL to 10 ng/mL induced more apoptosis in USP10-KO IVC-LSK cells than in USP10-WT cells. These results suggest that USP10-KO IVC-LSK cells are more sensitive to the loss of SCF, and that factors regulating SCF signaling could serve as a target for USP10-mediated inhibition of HSC apoptosis. Cell-surface c-KIT (SCF receptor) expression levels on USP10-KO and USP10-WT IVC-LSK cells were equivalent in both the presence and absence of HSC cytokines (Figure 6I), suggesting that c-KIT trafficking to the cell-surface membrane and c-KIT internalization in USP10-KO IVC-LSK cells are not altered by USP10 deletion.

We next attempted to identify a protein in IVC-LSK cells, whose expression is regulated by USP10 (Figure S3). USP10 stabilizes p53 and SIRT6 proteins by removing their K48-linked ubiquitin chains (Lin et al., 2013, Yuan et al., 2010). In addition, USP10 increases the expression of BECN1 and VPS34 proteins (Liu et al., 2011). However, the amount of p53, SIRT6, BECN1, and VPS34 proteins in USP10-KO IVC-LSK cells were equivalent to those of USP10-WT cells (Figure S3). We also measured the protein expression of several apoptosis-related genes in USP10-KO IVC-LSK cells. The amount of one pro-apoptotic BCL-2 family protein (PUMA) and three anti-apoptotic proteins (BCL-XL, BCL-2, and MCL-1) were slightly upregulated in USP10-KO cells. However, their role in apoptosis of USP10-KO LSK cells is unlikely since their upregulation in USP10-KO cells was minimal. There was no significant difference in the expression of other apoptosis-related proteins, specifically FOXO3A, p62, LC3, phosphoribosomal protein S6, phospho-AKT, and phospho-p65. These results suggest that USP10 inhibits apoptosis of HSPCs by deubiquitinating molecule(s) other than the ones analyzed here.

### The Cell-Cycle Profile and ROS Production in USP10-KO HSCs Are Normal

**Figure 7:**
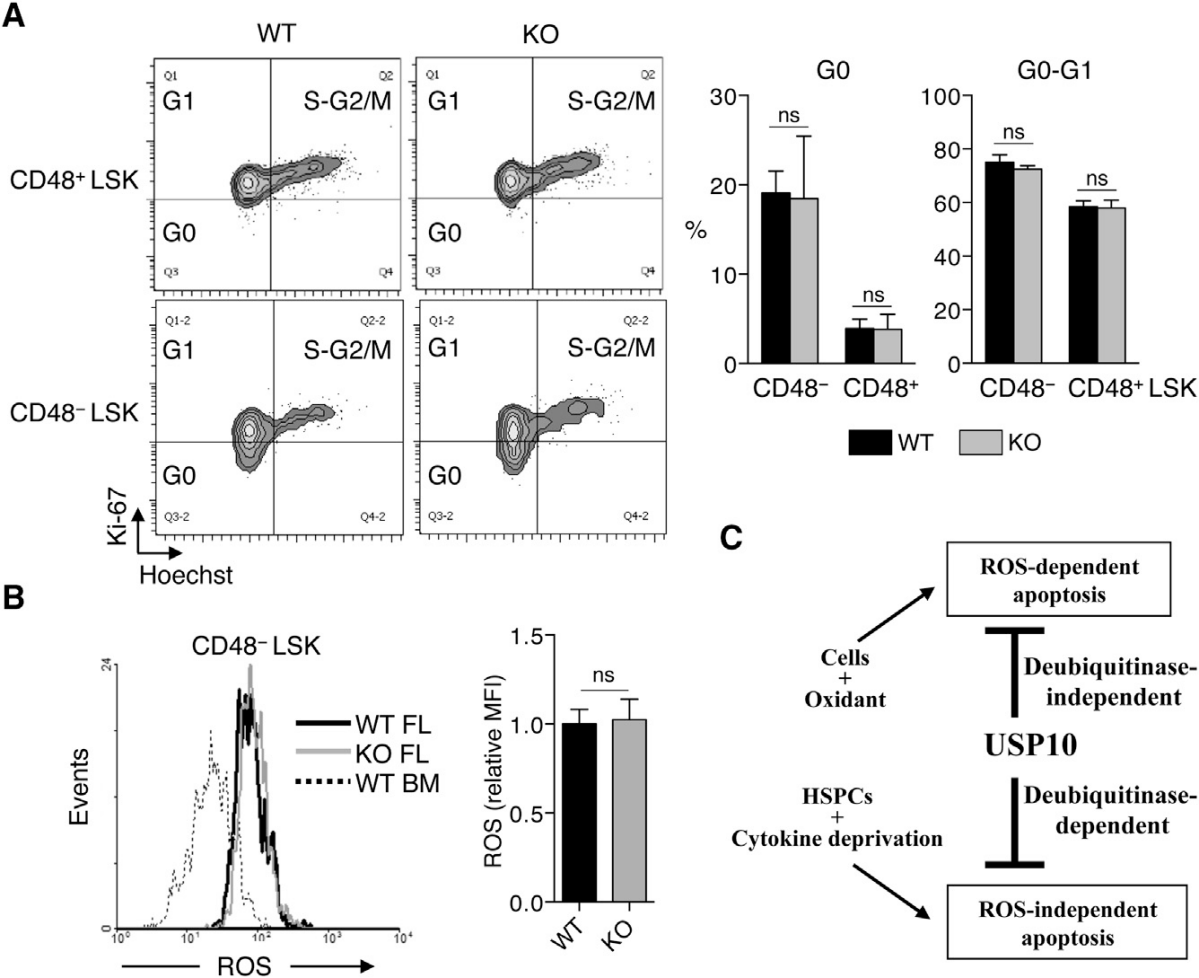
USP10-KO HSCs Demonstrate Both Normal Cell-Cycle Profile and ROS Production (A) Representative cell-cycle profile of CD48^−^ LSK and CD48^+^ LSK cells prepared from E14.5 USP10-WT and KO FL. The percentages of G0 and G0-G1 population in each cell fraction are shown in the right panels (mean ± SD; n = 7 and n = 5 for WT and KO, respectively). (B) ROS production in USP10-WT and KO FL HSCs. CD48^−^ LSK cells from E14.5 USP10-WT and KO FL were stained with CellROX Green reagent and analyzed by FACS. CD48^−^ LSK cells from 18-week-old WT BM were used as a control. A representative histogram and relative mean fluorescence intensities are shown (mean ± SD; n = 10 and n = 4 for WT and KO, respectively). (C) A schematic model for two distinct anti-apoptotic functions of USP10. While USP10 inhibits ROS-dependent apoptosis in a deubiquitinase-independent manner, it inhibits cytokine deprivation-induced apoptosis in HSPCs in a deubiquitinase-dependent manner. “n” represents number of embryos from which FL cells were prepared. ns, not significant.

It has been reported that FL HSCs actively undergo cell cycling but transiently enter into a quiescent G0 state. The transition to the quiescent state is critical for the maintenance of LT-HSCs in FL (Nygren et al., 2006). Thus, we analyzed the cell-cycle status of USP10-KO cells. The Ki-67-low quiescent G0 population was detected to a higher degree in CD48^−^ LSK (HSC/MPP) cells prepared from E14.5 FL compared with CD48^+^ LSK (HPC) cells, but the proportions of the G0 and G0-G1 population in USP10-KO cells were equivalent to those of USP10-WT cells (Figure 7A), thus indicating that USP10 loss in FL LSK cells (HSPCs) does not affect their cell-cycle status.

Augmented ROS production in HSCs is the frequent cause of HSC depletion in multiple gene-KO mice (Amrani et al., 2011, Chen et al., 2008, Ito et al., 2004, Liu et al., 2010, Miyamoto et al., 2007, Tothova et al., 2007). However, the ROS level in USP10-KO CD48^−^ LSK cells from E14.5 FL was equivalent to that of USP10-WT cells (Figure 7B). These results suggest that the enhanced apoptosis of USP10-KO HSCs and the defective hematopoiesis in USP10-KO mice are ROS independent. This is consistent with ROS-independent apoptosis inhibition by USP10 in IVC-LSK cells.

## Discussion

In this study, we found that systemic USP10-KO mice develop BM failure with pancytopenia including severe anemia, which is caused by the severe reduction of LT-HSCs (CD150^+^CD48^−^ LSK) in FL and BM. The BM transplantation assay indicated that the cell intrinsic defect of USP10-KO LT-HSCs was the main factor in the severe reduction of LT-HSCs in USP10-KO mice, because USP10-WT BM cells efficiently reconstituted hematopoiesis in USP10-KO mice. However, it should be noted that our data do not exclude the possibility that the cell extrinsic defect of USP10-KO mice also plays a role in the reduction of LT-HSCs in USP10-KO mice. Further analysis using hematopoietic cell-specific USP10-KO mice might provide a more definitive answer for this issue.

USP10-KO HSPCs in FL underwent more apoptosis than did USP10-WT HSPCs (Figures 5A and 5B). Moreover, USP10-KO HSPCs required higher in vitro concentrations of HSC cytokines, especially SCF, to inhibit apoptosis than did USP10-WT HSPCs (Figures 6D and 6H). These results suggest that the amount of SCF secreted in the FL niche is sufficient to inhibit apoptosis of USP10-WT but not USP10-KO HSPCs, thereby reducing the number of HSPCs in USP10-KO FL. Intriguingly, USP10-KO had little effect on the apoptosis of LK cells (LCPs). These findings suggest that the sensitivity of HSPCs—including LT-HSCs—to apoptosis is distinct from those of LCPs. Therefore, LCPs might be less dependent on SCF for survival than HSPCs.

A deubiquitinase-inactive USP10 mutant (USP10/C418A) did not inhibit cytokine deprivation-induced apoptosis of IVC-LSK cells, indicating that a factor deubiquitinated by USP10 inhibits apoptosis of IVC-LSK cells. We could not identify an anti-apoptotic molecule whose protein stability was increased by USP10 in IVC-LSK cells. Notably, of the HSC cytokines we used to expand and maintain IVC-LSK cells, SCF completely inhibited the apoptosis of IVC-LSK cells induced by deprivation of HSC cytokines. Furthermore, a lower concentration of SCF resulted in increased apoptosis in USP10-KO cells compared with USP10-WT cells. These results suggest that SCF signaling and its associated factors function in USP10-mediated inhibition of HSPC apoptosis. Since cell-surface expression levels of c-KIT on USP10-KO HSPCs were normal, downstream molecules involved in intracellular SCF signaling are likely to play a role in USP10-mediated inhibition of apoptosis.

Several deubiquitinases, such as USP1, USP3, USP16, and A20, are reported to be essential for proper HSC functions (Gu et al., 2016, Lancini et al., 2014, Nagamachi et al., 2014, Nakagawa et al., 2015, Parmar et al., 2010). For example, a lack of A20 in mice leads to defective HSC quiescence and reduced HSC numbers caused by enhanced NF-κB activation (Nagamachi et al., 2014, Nakagawa et al., 2015). Conversely, two ubiquitin ligases, c-CBL and ITCH, negatively regulate HSC functions because their KO in mice increases HSC numbers and enhances the hematopoietic reconstitution activity in a BM transplantation assay (Rathinam et al., 2008, Rathinam et al., 2011). Of particular interest is c-CBL, because it is a negative regulator of c-KIT signaling (Zeng et al., 2005). Thus, USP10 might deubiquitinate c-CBL substrate(s) and positively regulate c-KIT signaling.

Upon exposure to an oxidant, USP10 inhibits ROS-dependent apoptosis by reducing ROS production (Takahashi et al., 2013b). Since aberrant ROS production has been shown to be a critical apoptosis inducer of HSCs under basal and stress conditions (Naka et al., 2008), it was initially assumed that USP10 inhibits apoptosis of HSCs by inhibiting ROS production. However, our results using USP10 functional mutants indicated that ROS inhibition by USP10 is dispensable for USP10 to inhibit apoptosis of HSCs. These results indicate that USP10 inhibits apoptosis by two distinct mechanisms: one is deubiquitinase-independent/ROS-dependent and the other is deubiquitinase-dependent/ROS-independent. Furthermore, the latter inhibits cytokine deprivation-induced apoptosis of HSPCs including LT-HSCs in FL (Figure 7C).

Stress granules are stress-inducible cytoplasmic RNA granules containing RNAs and many RNA binding proteins (Kedersha et al., 2013). Upon exposure to an oxidant, USP10 is incorporated into stress granules by interacting with the RNA binding protein G3BP1 (Takahashi et al., 2013b). Studies using USP10 mutants, including USP10/77–792 and USP10/95–792, indicate that USP10's interaction with G3BP1 is essential for inhibition of ROS-dependent apoptosis and for USP10 incorporation into stress granules (Takahashi et al., 2013b). Unlike inhibition of oxidant-induced apoptosis, USP10 mutants lacking the ability to interact with G3BP1 (USP10/77–792, USP10/95–792) inhibited cytokine deprivation-induced apoptosis in FL IVC-LSK cells. Thus, USP10 interaction with G3BP1 and the incorporation of USP10 into stress granules are dispensable for USP10 inhibition of cytokine deprivation-induced apoptosis in FL HSPCs. Further mechanistic insights are needed to understand the precise role of USP10 in HSCs.

USP10-KO mice with a B6 genetic background died within 1 day of birth, but these USP10-KO mice were not anemic or pancytopenic. Thus, these early deaths did not result from complications of anemia. Therefore, USP10-KO mice with a B6 genetic background should possess another lethal abnormality distinct from hematopoietic failure.

## Experimental Procedures

### Mice

An institutional review committee at Niigata University and Institutional Animal Care and Use Committee of RIKEN Kobe Branch approved all mouse procedures. To obtain USP10-KO F2 hybrid mice, we crossed *Usp10*^Δ/+^ mice that had been backcrossed more than five times with B6 mice with BALB/cByJ mice (CLEA Japan), and their *Usp10*^Δ/+^ offspring were intercrossed with each other. To obtain USP10-KO F1 hybrid mice, we intercrossed *Usp10*^Δ/+^ mice that had been backcrossed more than eight times with B6 mice, or more than six times with BALB/cByJ mice (CLEA Japan), with each other. C57BL/6 CD45.1 congenic mice (B6-CD45.1) were obtained from Sankyo-Lab Service.

### Blood Cell Count

For analysis of blood counts, peripheral blood was collected from the tail vein with a heparinized hematocrit capillary (Hirschmann Laborgeräte) and analyzed on the hematology analyzer Celltac (Nihon Kohden).

### Cell Isolation

For preparation of single-cell suspensions of spleen and thymus, mouse spleen or thymus in minimum essential medium (MEM) supplemented with 2% fetal bovine serum (FBS) were mashed using a syringe plunger and passed through steel mesh. For preparation of BM cells, mouse femurs (for cell number counting) or vertebrates (for cell-surface staining) in MEM/2% FBS were crushed using a mortar and passed through 70-μm nylon mesh. RBCs were removed from spleen and BM samples by treating them with RBC lysis buffer (eBioscience). FL cells were collected at E14.5 or E17.5 and prepared by passing the FL through a 40-μm cell strainer (BD Biosciences) in DMEM containing 2% FBS. Unused FL cells were frozen using CryoScarless (BioVerde) for in vitro experiments.

### Flow Cytometry

For cell lineage analysis, BM and spleen cells were stained with the antibodies identified below. All staining was performed at 4°C in PBS with 2% FBS and 0.02% NaN_3_. Before staining, all samples were blocked with Fc blocking antibody (2.4G2). Propidium iodide (Sigma-Aldrich) was used to discriminate dead cells. The antibodies used were CD3e-fluorescein isothiocyanate (FITC), B220-FITC, TER-119-PE, B220-PE, MAC-1-FITC, and GR-1-PE. For analysis of HSCs, BM and FL cells were stained with the following antibodies: CD4-PerCP-Cy5.5, CD8a-PerCP-Cy5.5, CD3e-PerCP-Cy5.5, B220-PerCP-Cy5.5, GR-1-PerCP-Cy5.5, MAC-1-PerCP-Cy5.5, TER-119-PerCP-Cy5.5, SCA-1-PE-Cy7, CD150-FITC, CD48-PE, and c-KIT-APC. MAC-1 antibody was omitted for FL cells. For apoptosis detection, FL cells were first stained with PerCP-Cy5.5 lineage antibodies, SCA-1-PE-Cy7, c-KIT-APC, and CD48-PE, then stained with annexin V-FITC (BD Biosciences) and DAPI (Dojindo). For cell-cycle analysis, FL cells were first incubated with LIVE/DEAD fixable near-IR dye (Thermo Scientific) to discriminate dead cells. Cells were then stained with the PerCP-Cy5.5 lineage antibodies, SCA-1-PE-Cy7, c-KIT-APC, and CD48-PE, and were fixed and permeabilized using a FIX & PERM Kit (Thermo Scientific). The fixed cells were further stained with the Alexa Fluor 488 Ki-67 antibody and incubated with PBS containing 1 μg/mL RNase A (Nippongene), 0.05% saponin, and 20 μM Hoechst 33342 (Sigma-Aldrich). For ROS detection, freshly isolated BM or FL cells were incubated with 0.5 μM CellROX Green reagent (Thermo Scientific) at 37°C for 30 min and stained with the PerCP-Cy5.5 lineage antibodies, SCA-1-PE-Cy7, c-KIT-APC, and CD48-PE. For apoptosis detection in peripheral hematopoietic cells, splenic cells were stained with B220-PE and CD3e-PerCP-Cy5.5, or GR-1-PE and MAC-1-PerCP-Cy5.5, then incubated with 0.5 μM CellEvent Caspase-3/7 Green reagent (Thermo Scientific) at room temperature for 45 min. TER-119-APC was used to gate out RBCs. Details of the antibodies are listed in Table S1. Data collection was performed on a FACSCalibur or FACSAria II system (BD Biosciences) and data analysis was done using FACSDiva (BD Biosciences) or WinMDI software.

### BM and FL Cell Transplantation

For competitive hematopoietic reconstitution assay, CD45.2^+^ FL cells (5 × 10^5^) from USP10-WT or KO BALB/c/C57BL/6 F1 hybrid mice were mixed with competitor CD45.1^+^CD45.2^+^ BM cells (2 × 10^5^) from USP10-WT F1 hybrid mice, and the mixed cells were injected into the lateral tail vein of lethally irradiated (9.5 Gy) CD45.1^+^CD45.2^+^ WT F1 mice. Peripheral blood cells were sampled at multiple time points, RBC lysed, and stained with CD4-FITC, B220-FITC, MAC-1-PE, CD8a-FITC, CD19-PE, GR-1-PE, and CD45.1-APC to monitor multilineage hematopoiesis reconstitution by injected FL cells. CD45-PerCP-Cy5.5 was used to gate WBCs. For the hematopoiesis rescue assay, USP10-WT CD45.1^+^CD45.2^+^ BM cells (1 × 10^6^) were injected into 7- to 9-week-old CD45.2^+^ USP10-KO mice and the contribution of donor cells to peripheral blood cells was monitored by CD45.1 staining. Details of the antibodies are listed in Table S1.

### Cell Culture

FL cells were collected at E14.5, suspended in Iscove’s modified Dulbecco’s medium supplemented with 15% FBS, 100 U/mL penicillin G, 100 μg/mL streptomycin, 2 mM L-glutamine, 55 μM 2-mercaptoethanol, and HSC cytokines 50 ng/mL murine SCF, 50 ng/mL murine IL-6, 50 ng/mL human FLT3 ligand, 50 ng/mL human TPO, and 20 ng/mL murine IL-3 (all from PeproTech), and plated in an ultra-low attachment 24-well plate (Corning Costar). Cells were passaged at 2.5 × 10^5^/mL every 3 or 4 days. c-KIT^+^ cells were collected using the MACS cell separation method using mouse CD117 (c-KIT) microbeads (Miltenyi Biotec). CTLL-2, a mouse T cell line, was cultured in RPMI-1640 supplemented with 10% FBS, 100 U/mL penicillin G, 100 μg/mL streptomycin, 2 mM L-glutamine, 55 μM 2-mercaptoethanol, and 500 pM human IL-2.

### Colony-Formation Assay

In vitro cultured FL cells (1 × 10^4^) were plated in 1 mL of MethoCult M3434 medium (Stem Cell Technologies) on a 35-mm plate, cultured at 37°C for 14 days, and examined for colony formation.

### Isolation of Mouse Usp10 cDNA

Total RNA was isolated from CTLL-2 cells with RNAiso (Takara), and cDNA was synthesized with oligo(dT) primer and SuperScript III reverse transcriptase (Thermo Scientific). The open reading frame of mouse Usp10 cDNA was PCR-amplified and cloned into pENTR/D-TOPO (Thermo Scientific).

### Plasmids

For construction of a bicistronic lentiviral vector expressing blasticidin deaminase (Bsd) and mouse USP10, Bsd was first PCR-amplified from CSII-CMV-MCS-IRES2-Bsd (provided by Dr. H. Miyoshi of RIKEN Tsukuba Institute) and inserted into the BamHI and NotI sites of pMXs-IG (provided by Dr. T. Kitamura of Tokyo University). The GFP sequence downstream of the internal ribosome entry site (IRES) was replaced with mouse Usp10 cDNA using PCR. A Bsd-IRES-Usp10 fragment was cloned into the SalI and XhoI sites of the Gateway entry vector pENTR 2B (Thermo Scientific). Entry vectors for Usp10 mutants (77–792, 95–792, and C418A) were constructed by replacing Usp10-WT in pENTR-Bsd-IRES-Usp10 with the respective mutants using PCR. pENTR-Bsd-IRES-GFP was constructed by inserting a Bsd-IRES-GFP fragment into the SalI and XhoI sites of pENTR 2B. CS-CMV-RfA is a lentiviral Gateway destination vector constructed by inserting Gateway reading frame cassette A into the NheI and HpaI sites of CSII-CMV-MCS-IRES2-Bsd. Bsd-IRES-Usp10, Bsd-IRES-Usp10 mutants, or Bsd-IRES-GFP was transferred to CS-CMV-RfA by an LR recombination using LR Clonase (Thermo Scientific).

### Lentiviruses

pCAG-HIVgp, pCMV-VSV-G-RSV-Rev (provided by Dr. Miyoshi), and the respective lentiviral vectors were transfected into 293T cells using FuGENE HD (Promega) to generate recombinant lentiviruses. The supernatant of the 293T cells containing the lentivirus was concentrated using Amicon Ultra-15 Filter Units (EMD Millipore) and used to infect in vitro cultured FL cells. Cells were cultured in the medium containing 6 μg/mL blasticidin (Thermo Scientific) for about 1 week to establish stably infected FL cells.

### Statistical Analysis

Statistical analysis was performed with GraphPad Prism software (GraphPad). The nonparametric Mann-Whitney U test was used for statistical comparisons unless otherwise indicated.

## Author Contributions

M.H. designed the study, performed experiments, analyzed and interpreted data, and wrote the manuscript; H. Kawamura designed the study, performed experiments, and analyzed and interpreted data; H.M. and H. Kiyonari performed experiments; T.H., M.T., S.S., and K.S. assisted in performing experiments; S.J. and M.N. performed experiments and interpreted data; M.F. designed the study, interpreted data, and wrote the manuscript.

## Supplemental Information

Document S1. Supplemental Experimental Procedures, Figures S1–S3, and Table S1

Document S2. Article plus Supplemental Information
