# Supplementary material for: USP10 Is an Essential Deubiquitinase for Hematopoiesis and Inhibits Apoptosis of Long-Term Hematopoietic Stem Cells
Source: Stem Cell Reports. 2016 Dec 13;7(6):1116–29. doi: 10.1016/j.stemcr.2016.11.003 (PMC5161743; doi:10.1016/j.stemcr.2016.11.003)
Supplement: Document S1. Supplemental Experimental Procedures, Figures S1–S3, and Table S1 [file mmc1.pdf]

Stem Cell Reports, Volume 7

Supplemental Information

USP10 Is an Essential Deubiquitinase for Hematopoiesis and Inhibits Apoptosis of Long-Term Hematopoietic Stem Cells

Masaya Higuchi, Hiroki Kawamura, Hideaki Matsuki, Toshifumi Hara, Masahiko Takahashi, Suguru Saito, Kousuke Saito, Shuying Jiang, Makoto Naito, Hiroshi Kiyonari, and Masahiro Fujii

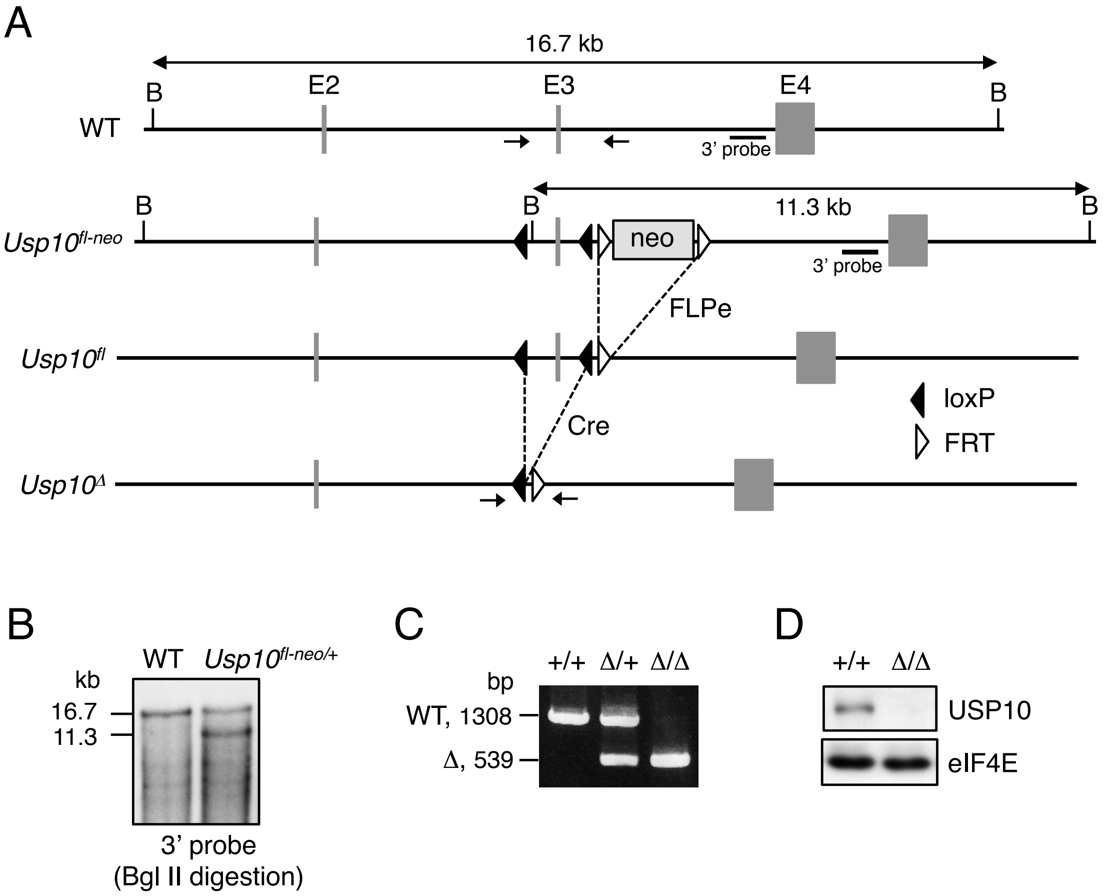

Figure S1 related to figure 1. Generation of USP10-KO mice. (A) Genomic structure of mouse *Usp10*. Grey rectangles (E2, E3, E4) represent *Usp10* exons. Neo indicates the Neomycin selection cassette flanked by FRT recombination sites (white triangles), inserted between exon 3 and 4. Black triangles represent the loxP sites, flanking exon 3. Removal of the Neomycin cassette by crossing with *CAG-FLPe* mice resulted in the generation of the conditional *Usp10*^{fl} allele. The conditional *Usp10*^{fl} allele was converted to the recombined *Usp10*$^{\Delta}$ allele by crossing with *TLCN-Cre* mice. Positions of primers used for genotyping are indicated as small arrows. The Bgl II recognition sites were indicated as B. (B) Southern blot analysis of the tail DNA derived from *Usp10*$^{fl\text{-}neo/+}$ heterozygous mice. (C) PCR genotyping of USP10-KO mice. The primer sequences are 5'-GGTGTTTGGGGCTCGGTTCTGTCA-3' and 5'-TGGCAGTTGTGGTGGTTTGAGTATG-3'. (D) USP10 protein expression in USP10-WT and KO MEFs. Total cell lysates were prepared from USP10-WT and KO MEFs and probed with anti-USP10 or anti-eIF4E antibody.

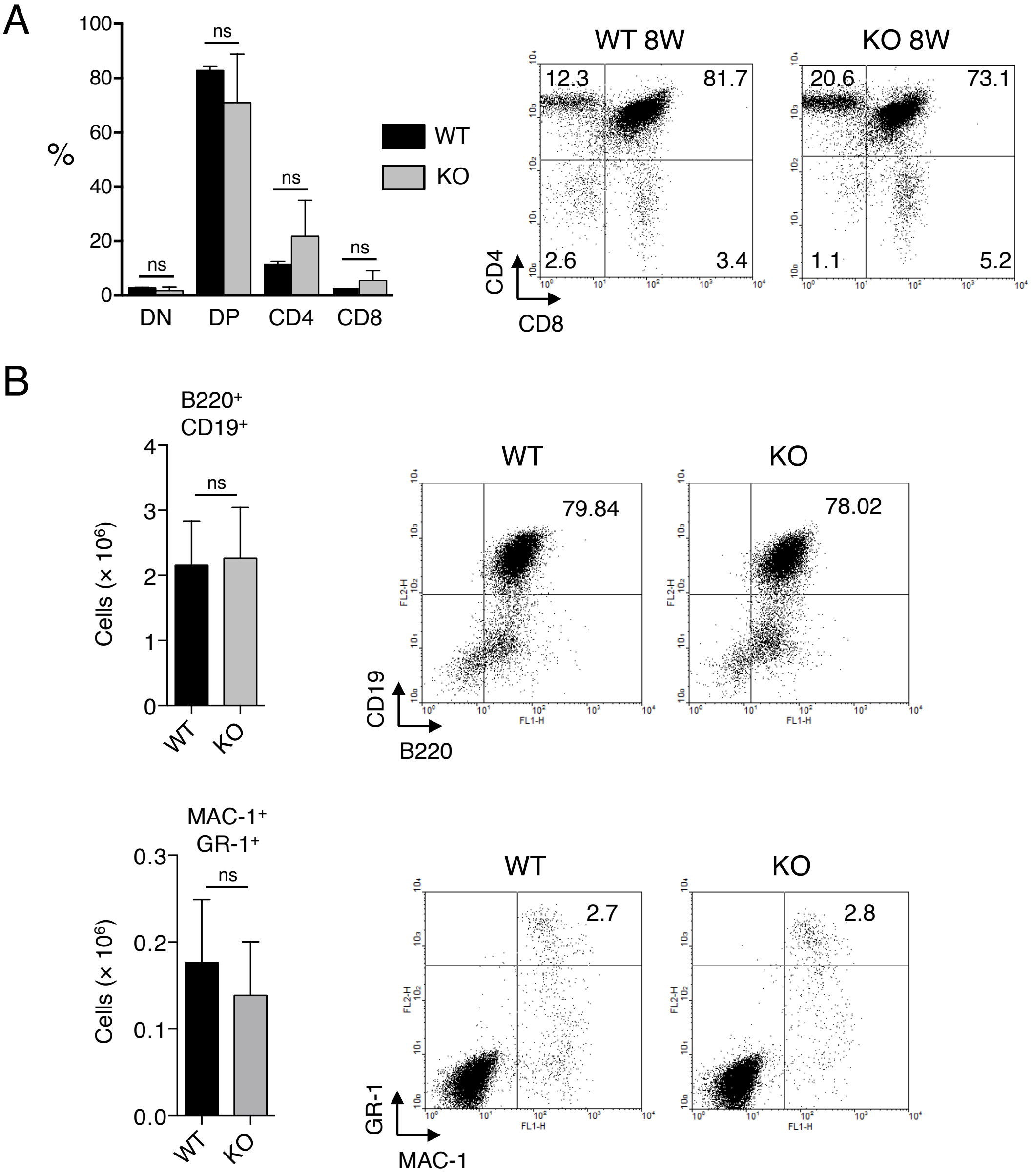

Figure S2 related to figure 2. (A) T cell development in 8-week-old USP10-WT and KO mice (mean ± SD; n = 5 for each genotype). A representative FACS analysis of thymocytes from 8-week-old USP10-WT and KO mice is shown. Numbers indicate the percentages of each population. (B) Differentiation of USP10-WT and KO FL c-KIT$^+$ progenitor cells into B and myeloid cells *in vitro*. FL c-KIT$^+$ cells were plated on OP9 cells and cultured in the presence of IL-7 and FLT3-ligand for 6 days. The total number of B220$^+$CD19$^+$ and MAC-1$^+$GR-1$^+$ cells and a representative FACS analysis of differentiated cells are shown (mean ± SD; n = 3 for each genotype). Numbers indicate the percentages of each population. “n” represents number of embryos from which c-KIT$^+$ cells were prepared.

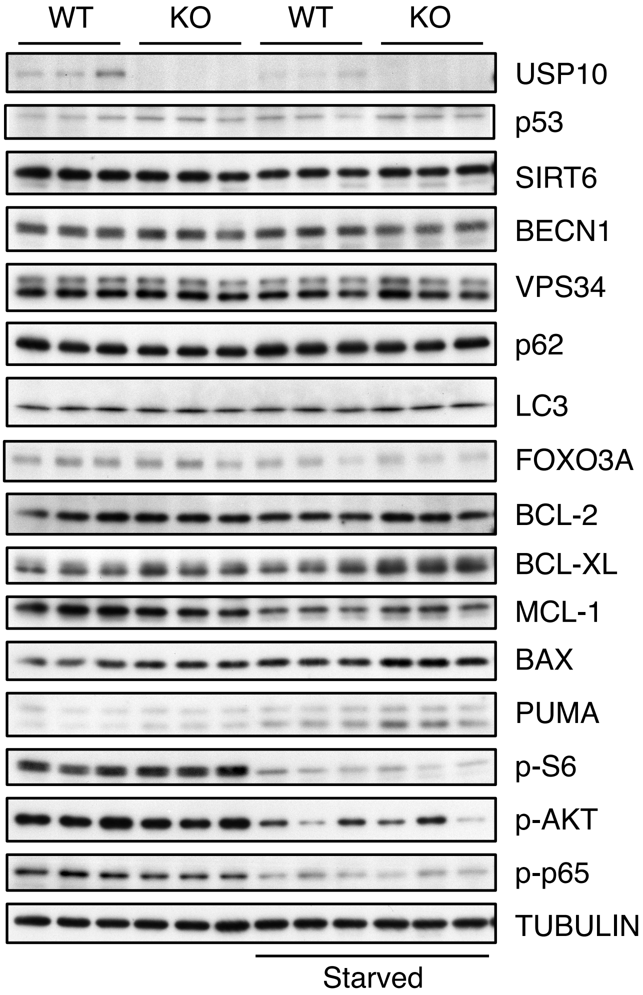

Figure S3 related to figure 6. Total cell lysates from USP10-WT and KO IVC- LSK cells in the presence of HSC-cytokines or after starvation for 24 h were analyzed by western blotting using the indicated antibodies.

Antibody	Clone	Vendor	Catalogue #
B220-FITC	RA3-6B2	BD Biosciences	11-0452
B220-PE	RA3-6B2	eBiosciences	12-0452
B220-PerCP-Cy5.5	RA3-6B2	eBioscience	45-0452
CD3e-FITC	145-2C11	BD Biosciences	561827
CD3e-PerCP-Cy5.5	145-2C11	eBioscience	45-0031
CD4-FITC	GK1.5	BD Biosciences	553729
CD4-PE	RM4-5	eBioscience	12-0042
CD4-PerCP-Cy5.5	RM4-5	eBioscience	45-0042
CD8a-FITC	53-6.7	eBioscience	11-0081
CD8a-PerCP-Cy5.5	53-6.7	eBioscience	45-0081
CD19-PE	eBio1D3	eBioscience	12-0193
CD45-PerCP-Cy5.5	30-F11	eBioscience	45-0451
CD45.1-APC	A20	eBioscience	17-0453
CD48-PE	HM48-1	eBioscience	12-0481
CD150-FITC	9D1	eBioscience	11-1501
c-KIT-APC	2B8	eBioscience	17-1171
Ki-67-Alexa Fluor 488	B56	BD Biosciences	558616
GR-1-PE	RB6-8C5	eBioscience	12-5931
GR-1-PerCP-Cy5.5	RB6-8C5	eBioscience	45-5931
MAC-1-FITC	M1/70	eBioscience	11-0112
MAC1-PE	M1/70	BD Biosciences	553311
MAC-1-PerCP-Cy5.5	M1/70	eBioscience	45-0112
SCA-1-PE-Cy7	D7	eBioscience	25-5981
TER-119-PE	TER-119	BD Biosciences	553673
TER-119-APC	TER-119	BD Biosciences	561033
TER-119-PerCP-Cy5.5	TER-119	eBioscience	45-5921

Table S1 related to Experimental Procedures. List of antibodies used for flow cytometric analyses.

SUPPLEMENTAL EXPERIMENTAL PROCEDURES

Mice

An institutional review committee at Niigata University and Institutional Animal Care and Use Committee of RIKEN Kobe Branch approved all mouse procedures. Conditional USP10-KO mice (Accession No. CDB0605K: http://www2.clst.riken.jp/arg/mutant%20mice%20list.html) were generated by homologous recombination in an embryonic stem cell line (TT2) (Yagi et al., 1993) using a gene targeting vector, in which exon 3 of the mouse genomic *Usp10* DNA fragment was flanked by *loxP* sites (Figure S1A). The details of the gene targeting strategy can be provided upon request. Embryonic stem cell clones carrying the Cre recombinase-deletable *Usp10* allele (*fl-neo*) were used to generate chimeric mice. After germline transmission of the $Usp10^{fl\text{-}neo/+}$ allele, $Usp10^{fl\text{-}neo/+}$ heterozygous mice were obtained (Figure S1B). The offspring were further crossed with FLPe recombinase transgenic mice, which excises the neomycin resistant gene cassette (*neo*) by FLPe recombinase generating heterozygous floxed USP10 ($Usp10^{fl/+}$) mice. *CAG-FLPe* mice (Kanki et al., 2006) were obtained from Riken BRC (Tsukuba, Japan). $Usp10^{fl/+}$ mice were bred with the telencephalin (*TLCN*)-*Cre* mice carrying the *Cre* recombinase gene regulated by the endogenous *TLCN* gene promoter (Fuse et al., 2004). In the *TLCN-Cre* mice, Cre recombinase is expressed at the post-implantation stage, thereby enabling the excision of the floxed DNA fragment in germ cells. *TLCN-Cre* mice were generous gifts from Dr. Masayoshi Mishina (The University of Tokyo). Mice with the germ line deletion of the *Usp10* exon 3 ($Usp10^{\Delta/+}$) were backcrossed to C57BL/6J (B6) mice (CLEA Japan) more than three times, and systemic $Usp10^{\Delta/\Delta}$ (USP10-KO) mice were obtained by intercrossing $Usp10^{\Delta/+}$ (USP10-HET) mice with each other (Figure S1C). The excision of the *Usp10* exon 3 induces a frame shift mutation of USP10 protein to generate a short peptide consisting of the first 30 amino acids of USP10 polypeptide and an unrelated 10 amino acids. MEFs were established using USP10-WT and KO E14.5 embryos as described previously (Takahashi et al., 2013b), and USP10 protein was undetectable in USP10-KO MEFs (Figure S1D).

T cell differentiation analysis

Thymocytes from 8-week-old USP10-WT and KO mice were stained with CD4-PE and CD8-FITC antibodies and analyzed using a FACSCalibur (BD Biosciences) and WinMDI software. Propidium iodide (Sigma-Aldrich) was used to discriminate dead cells.

In vitro differentiation of FL HSPCs

OP9 cells were obtained from Riken BRC and cultured in a-MEM supplemented with 20% FBS, 100 U/ml penicillin G, 100 μg/ml streptomycin, 2 mM L-glutamine, and 55 μM 2-ME. One day before plating FL HSPCs, OP9 cells were treated with 10 μg/ml Mitomycin C (Wako) for 2 h and plated to a 24-well plate. c-KIT$^+$ cells were collected from E14.5 USP10-WT and KO FL cells using mouse CD117 (c-KIT) microbeads (Miltenyi Biotec). Next, 5×10^3 c-KIT$^+$ cells were suspended in 1 ml IMDM supplemented with 15% FBS, 100 U/ml penicillin G, 100 μg/ml streptomycin, 2 mM L-glutamine, 55 μM 2-mercaptoethanol, 10 ng/ml murine IL-7, and 5 ng/ml human FLT3-ligand (PeproTech), and plated onto Mitomycin C-treated OP9 cells. After culturing for 6 days, cells were harvested using Accutase (Innovative Cell Technologies), Fc-blocked by 2.4G2, stained with B220-FITC and CD19-PE or MAC-1-FITC and GR-1-PE, and analyzed using a FACSCalibur and WinMDI software. Propidium iodide was used to discriminate dead cells.

Western blotting

To prepare total cell extracts, cells were lysed in SDS sample buffer (2% SDS, 62.5 mM Tris-HCl pH 6.8, 10% glycerol, 50 mM dithiothreitol, 0.01% bromophenol blue) and heated at 95°C for 5 min. They were size-separated by SDS-PAGE, transferred onto a polyvinylidene difluoride membrane (Bio-Rad Laboratories), and probed with following antibodies: α-TUBULIN (CP06; Calbiochem), BAX (2772; Cell Signaling Technology), BCL-2 (2870; Cell Signaling Technology), BCL-XL (610746; BD Biosciences), BECN1 (sc-11427; Santa Cruz Biotechnology), eIF4E (9742; Cell Signaling Technology), FOXO3A (2497; Cell Signaling Technology), LC3 (PM036; MBL), MCL-1 (5453; Cell Signaling Technology), p53 (sc-99; Santa Cruz Biotechnology), p62/SQSTM1 (PM045; MBL), Phospho-AKT (Ser473) (2336; Cell Signaling Technology), Phospho-S6 Ribosomal Protein (Ser235/236) (4857; Cell Signaling Technology), PI3 Kinase Class III (4263; Cell Signaling Technology), PUMA (7467; Cell

Signaling Technology), SIRT6 (12486; Cell Signaling Technology), and USP10 (5553; Cell Signaling Technology).

SUPPLEMNTAL REFERENCES

Fuse, T., Kanai, Y., Kanai-Azuma, M., Suzuki, M., Nakamura, K., Mori, H., Hayashi, Y., and Mishina, M. (2004). Conditional activation of RhoA suppresses the epithelial to mesenchymal transition at the primitive streak during mouse gastrulation. Biochem. Biophys. Res. Commun. *318*, 665-672.

Kanki, H., Suzuki, H., and Itohara, S. (2006). High-efficiency CAG-FLPe deleter mice in C57BL/6J background. Exp. Anim. *55*, 137-141.

Yagi, T., Tokunaga, T., Furuta, Y., Nada, S., Yoshida, M., Tsukada, T., Saga, Y., Takeda, N., Ikawa, Y., and Aizawa, S. (1993). A novel ES cell line, TT2, with high germline-differentiating potency. Anal. Biochem. *214*, 70-76.
